# Specificity and tunability of efflux pumps: A new role for the proton gradient?

**DOI:** 10.1371/journal.pcbi.1012772

**Published:** 2025-01-27

**Authors:** Matthew Gerry, Duncan Kirby, Boian S. Alexandrov, Dvira Segal, Anton Zilman

**Affiliations:** 1 Department of Physics, University of Toronto, Toronto, Ontario, Canada; 2 Theoretical Division, Los Alamos National Laboratory, Los Alamos, New Mexico, United States of America; 3 Chemical Physics Theory Group, Department of Chemistry and Centre for Quantum Information and Quantum Control, University of Toronto, Toronto, Ontario, Canada; Shiraz University, IRAN, ISLAMIC REPUBLIC OF

## Abstract

Efflux pumps that transport antibacterial drugs out of bacterial cells have broad specificity, commonly leading to broad spectrum resistance and limiting treatment strategies for infections. It remains unclear how efflux pumps can maintain this broad spectrum specificity to diverse drug molecules while limiting the efflux of other cytoplasmic content. We have investigated the origins of this broad specificity using theoretical models informed by the experimentally determined structural and kinetic properties of efflux pumps. We developed a set of mathematical models describing operation of efflux pumps as a discrete cyclic stochastic process across a network of states characterizing pump conformations and the presence/absence of bound ligands and protons. These include a minimal three-state model that lends itself to clear analytic calculations as well as a five-state model that relaxes some of the simpler model’s most strict assumptions. We found that the pump specificity is determined not solely by the drug affinity to the pump–as is commonly assumed–but it is also directly affected by the periplasmic pH and the transmembrane potential. Therefore, changes to the proton concentration gradient and voltage drop across the membrane can influence how effective the pump is at extruding a particular drug molecule. Furthermore, we found that while both the proton concentration gradient across the membrane and the transmembrane potential contribute to the thermodynamic force driving the pump, their effects on the efflux enter not strictly in a combined proton motive force. Rather, they have two distinguishable effects on the overall throughput. These results highlight the unexpected effects of thermodynamic driving forces out of equilibrium and illustrate how efflux pump structure and function are conducive to the emergence of multidrug resistance.

## Author summary

Efflux pumps play an important role in the ability of bacteria to develop resistance to a broad range of antibiotics by effectively pumping out a variety of different drug molecules. While maintaining a broad range of molecular targets, the pump must exhibit enough specificity to avoid pumping out molecules necessary for cell survival. We have explored this phenomenon through kinetic modeling of a generic proton-powered efflux pump inspired by experiments on Resistance-Nodulation-Division pumps found in gram-negative bacteria. This modeling indicates that the specificity of efflux pumps is influenced not only by equilibrium molecular interactions but also by nonequilibrium effects involving the proton concentration gradient and the membrane electric potential.

## Introduction

In recent decades, bacterial antibiotic resistance has emerged as a major and pervasive threat to public health and, consequently, it has become a critical subject of study of high biomedical significance [1–4]. Bacterial drug resistance is a complex, dynamic response to the evolutionary pressure generated by the widespread use of antibiotics in modern society [3,4]. In other words, bacteria rapidly evolve, predominantly via horizontal gene transfer [5,6], to diversify and optimize their defense mechanisms and to neutralize new antimicrobial agents [7]. Multidrug transmembrane active transporters, commonly known as bacterial efflux pumps, play a central role among these defense mechanisms. These pumps are effective against a wide range of different drugs, while at the same time exhibiting the specificity needed to preferentially extrude drug molecules, rather than self molecules, from the cell [7–13].

In this study, we have focused on minimal models for active transmembrane transporters, or efflux pumps, that are powered by proton concentration gradients across the inner membrane of the cell and can pump a variety of different drug molecules. In doing so, they confer multidrug resistance to bacterial cells. The models we considered are inspired loosely by various families of transporters known to be associated with multidrug resistance [11]. Resistance-Nodulation-Division (RND) efflux pumps comprise one such family, which includes the AcrAB-TolC pump found in *Escherichia coli* and the similarly structured MexAB-OprM pump found in *Pseudomonas aeruginosa*. RND pumps are elaborate protein complexes with a tripartite structure giving rise to a “rotary” pumping mechanism [14–19]. The efflux of antibiotics including nitrocefin and other cephalosporins through the AcrAB transporter have been measured in experiments [20]. Another relevant class of pumps consists of symporters and antiporters of the Major Facilitator Superfamily (MFS), which instead exhibit an alternating-access mechanism [21]. To study the general properties of efflux drug specificity, efficiency, and tunability common across different transporter classes, we subsume many of these molecular features into simpler, more abstract models whose kinetics can nevertheless serve as a good approximation to those of these more realistic pumps [22]. Conclusions drawn from these models also bear on the understanding of the operation of other antiporter pumps, involved in, for example, maintaining proton and sodium ion concentration gradients across membranes [23–25].

**Fig 1:**
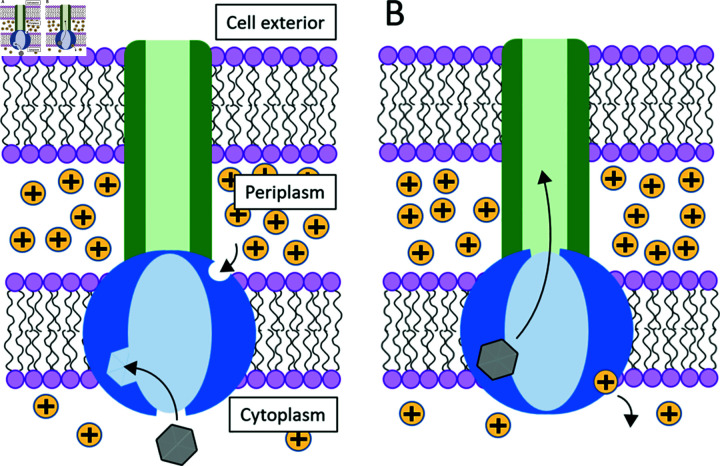
Schematic depiction of the proton antiporter pump. The pump considered in this work depicted in each of its two conformational states. The active component of the pump is in blue, coupled to the passive efflux channel shown in green. Arrows indicate the typical motion of ligands throughout the pumping process. A: A drug molecule (gray) binds from the cytoplasm to a binding site on the pump interior. A proton binds to its respective binding site, initially facing the proton-rich periplasm. B: Upon proton translocation toward the cytoplasm, the channel opens to the passive transporter (green) which allows the drug to exit the cell.

Schematics of pump operation are depicted in Fig 1. The pump spans the inner and outer membrane of the cell, with two functionally and spatially distinct components. The outer-membrane component is a passive exit conduit responsible for the diffusion of the antimicrobial agents into the extracellular space [26]. The inner-membrane component is responsible for the active pumping of the antimicrobial agents out of the cytoplasm via coupling to the flux of the protons. This flux is, in turn, driven by electric and chemical potential gradients across the membrane, which are present due to the relatively high concentration of protons within the periplasm, or intermembrane space. In light of this proton flux, it is implicit that other processes in the cell must dissipate energy in order to maintain these nonequilibrium gradients. This amounts to a thermodynamic cost the cell must pay to ensure that the pump preferentially delivers drug molecules from the cytoplasm towards the exit duct, regardless of the direction of the concentration gradient of the drug molecules themselves.

We considered two mathematical models of progressing complexity that describe the operation of the active inner-membrane transporter as a Markov jump process on a network of states. Transitions correspond to the binding and unbinding of ligands and changes in pump conformation. The use of such models demands the identification of a finite set of coarse-grained states approximating the operation of the system, while in reality, state evolution is a continuous process. These states are typically taken to represent the most stable and long-lived molecular conformations as determined through structural measurements. Numerous studies have employed models of this kind to describe the operation of various protein complexes, including ATP-powered pumps [27–29], molecular motors [30–33], ATP synthases [34,35], kinetic proofreading receptors [36,37], and DNA polymerases [38,39].

Our aim was to shed light on how an efflux pump can effectively transport a wide range of drug molecules with differing properties while simultaneously exhibiting the specificity needed to avoid pumping out the molecules crucial for the normal functioning of the cell. We have shown that, as expected, the periplasmic pH, the membrane potential, and the strength of proton and drug binding to the transporter all play a role in modulating the flux throughput of the pump. Notably, unlike the expectations based on Onsager theory close to equilibrium [40], the membrane potential and the pH modulate the pump throughput not strictly through their combination in the electrochemical potential of protons (broadly known as the proton motive force), but with each having their own distinct effect. This finding builds upon a similar distinction between the effects of the different proton motive force components derived from studies of bacterial flagella [30,31].

Furthermore, varying membrane potential and periplasmic pH parameters impacts not only the overall efflux rate, but also pump specificity, by shifting the range of drug binding affinities at which the pump throughput is maximized. This implies the capacity for an efflux pump to optimally extrude a range of different drug molecules under different conditions, and thus may play a role in explaining the hitherto puzzling broad specificity. In light of these findings, we discuss the functional cycle of the transporter and the coupling of its specificity and efficiency with the proton gradient [7].

This paper is structured as follows. The minimal three-state kinetic model of efflux pumps we developed is introduced and described in the Results section below. We assessed the different factors affecting the overall efflux as predicted by the model, in particular the nontrivial dependence of the efflux on the proton concentrations in the periplasm and cytoplasm, and on the membrane electric potential. We then considered how changes to the periplasmic pH can affect the range of drug binding affinities at which the pump is most effective, underlying the flexibility of efflux pumps to be able to play a role in the response to a variety of different drugs.

We also introduced a richer kinetic model that exemplifies the same effects, broadening the class of systems to which this analysis may apply. The Discussion expands on how our work highlights nontrivial effects seen in cellular processes taking place far from equilibrium, while fitting into the context of the existing understanding of multidrug resistance.

## Results

### Three-state kinetic model

We considered a simple model for a proton antiporter pump as depicted in Fig 1, with one binding site for the drug molecule initially facing the interior of the cell and one binding site for protons facing the periplasm. An analogous pump with multiple proton binding sites could be considered as a straightforward generalization. After the sequential binding of the drug molecule and the proton [19], a conformational change occurs in the pump as the proton is pulled by the electric potential gradient across the inner membrane from the periplasmic side towards the cytoplasmic side, coupled with the release of the drug into the cytoplasm. This exposes the ligands to their respective destinations: the drug molecule is able to unbind and be released to a passive channel that carries it out of the cell, and the protons are released to the cytoplasm.

**Fig 2:**
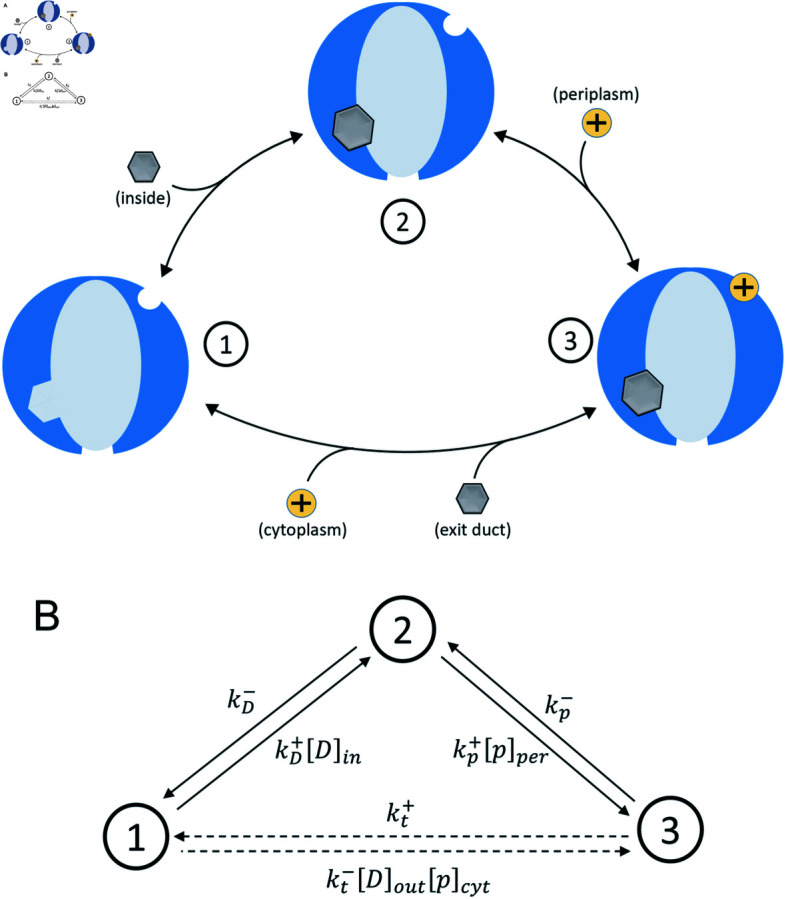
Diagrams of the kinetic model. A: Three-state kinetic model of the efflux pump, with reversible transitions represented by black arrows. Drug molecules and protons are shown where they bind to (unbind from) the pump, labelled with the relevant reservoir in each case. The forward process proceeds in the clockwise direction. B: The model represented in terms of directed transitions of a network of states, with associated transition rates shown. The mathematical formulation of the model is presented in Equation (1) and the kinetic parameters are defined in Equations (2)–(5).

To model the operation of this pump, we considered a three-state kinetic scheme, as shown in Fig 2A. The associated process characterizing one completion of the cycle depicted consists of three steps. As required for microscopic thermodynamic consistency, each of these steps can occur in either direction. Note that in the figure, the source (destination) of each ligand upon binding (unbinding) is denoted in parentheses.

In state 1, neither a drug molecule nor any protons are bound. Then, in a transition to state 2, a drug molecule binds to its respective binding site. We consider a pump with drug binding from the cytoplasm, as depicted in Fig 2, but we note that a proton antiporter pump like this may have multiple different drug binding sites associated with different drug molecular mass ranges. Drug binding may, in actuality, occur from either the cytoplasm or the periplasm [41–43]. Indeed, binding from the periplasm is believed to be most common for the AcrAB efflux pump of *E. coli*. [44,45]. Subsequently, a proton binds to its binding site taking the pump to state 3. The third and final step, which takes the pump back to its initial state, consists of four key sub-processes. Firstly, a conformational change occurs, closing the pump off to the interior of the cell and opening it to the exit duct, while the proton is simultaneously transported across the inner membrane towards the cytoplasm. Then the drug unbinds and is released to the exit duct. The proton unbinds and exits into the cytoplasm. Finally, in the absence of bound ligands, the pump returns to its original conformation, with the drug binding site exposed to the cell interior. Despite using an alternating-access mechanism rather than a rotary mechanism, this sequence of transitions between of coarse-grained states is motivated by experiments on pumps of the RND family [19]. However, the model itself is meant to be a simplified idealized representation of a broad class of transporters.

The simplicity of this model allowed us to obtain analytic results pertaining to the behaviour of efflux pumps, and it means that relatively few specific details were imposed, with hopes of achieving a large degree of generality. However, this simplicity is also the source of this model’s limitations. For instance, it assumes that multiple events always occur in tandem comprising the final 3 → 1 transition. It also potentially neglects additional steps that may be required as part of the pumping process, for instance, the transition of the drug molecule from the access pocket to the deep binding pocket characteristic of RND pumps. Relaxing various combinations of these constraints by adding states (and correspondingly expanding the space of parameters characterizing the kinetics), may lead to models that describe the operation of certain specific efflux pumps with greater accuracy, at the expense of mathematical tractability and generality. We explored this notion, as discussed below.

The system is described by the probabilities, *P*_1_, *P*_2_, and *P*_3_, of the transporter to be in each of the three states, which are governed by the corresponding master equation that describes the transitions between states. This approach towards modeling the system assumes that all relevant details can be captured by the values of a limited set of transition rates. These would include, for example, structural features of the particular drug molecule in question and any other factors affecting the affinity with which it binds to the pump. Conceptually similar models have been widely and successfully used in the past to describe active transporters [25,46–51]. Accordingly,


$$\begin{array}{llll} {Ṗ}_{1} & =-(k_{D}^{+}{\left[D\right]}_{in}+k_{t}^{-}{\left[D\right]}_{out}{\left[p\right]}_{cyt})P_{1}+k_{D}^{-}P_{2}+k_{t}^{+}P_{3}\; & & \;\\ {Ṗ}_{2} & =k_{D}^{+}{\left[D\right]}_{in}P_{1}-(k_{D}^{-}+k_{p}^{+}{\left[p\right]}_{per})P_{2}+k_{p}^{-}P_{3}\; & & \;\\ {Ṗ}_{3} & =k_{t}^{-}{\left[D\right]}_{out}{\left[p\right]}_{cyt}P_{1}+k_{p}^{+}{\left[p\right]}_{per}P_{2}-(k_{p}^{-}+k_{t}^{+})P_{3},\; \end{array}$$ (1)

where $k_{D}^{+}$ and $k_{D}^{-}$ are forward and reverse rate constants for the drug binding step, $k_{p}^{+}$ and $k_{p}^{-}$ are those for the proton binding step, and $k_{t}^{+}$ and $k_{t}^{-}$ are those for the final, combined step. [D]_*in*_ and [p]_*per*_ are the cytoplasmic concentration of the drug and periplasmic concentration of the protons, respectively (-log_10_[p]_*per*_ is the periplasmic pH). The master equation is represented graphically in Fig 2B, where each transition is labelled with its respective rate. Rates in Eqs. (1) for transitions that involve drug or proton binding are proportional to the relevant ligand concentration, following a law of mass action [52]. We took the rate at which the pump transports drug molecules and protons to be slow in comparison to other translocation processes happening in the cell, allowing concentrations to be assumed constant.

[D]_*out*_ and [p]_*cyt*_ are the extracellular drug concentration and cytoplasmic proton concentration. These factor into the transition rates for the backwards operation of the pump, which is rare under typical conditions, due to the electric potential gradient playing the strongest role in determining the directionality of pump operation.

The transition rates are expressed in terms of parameters characterizing the system. For the drug binding step, the ratio of the binding and unbinding rate constants is equal to the dissociation constant describing the drug interaction with the pump:


$$\begin{array}{c} \frac{k_{D}^{-}}{k_{D}^{+}}=K_{D}=\frac{1}{\nu_{D}}e^{E_{D}∕k_{B}T}, \end{array}$$ (2)

where *k*_*B*_ is the Boltzmann constant, *T* is the temperature of the surroundings, *E_D_* < 0 is the drug binding energy to the pump, and ν_*D*_ is a constant related to the volume and shape of the interacting moieties as well as the reference concentration used for chemical potential calculations.

Similarly, for the interaction of the protons with the protonatable groups in the inner-membrane domain:


$$\begin{array}{c} \frac{k_{p}^{-}}{k_{p}^{+}}=K_{P}=\frac{1}{\nu_{p}}e^{E_{p}∕k_{B}T}. \end{array}$$ (3)

The constant *K_p_* is linked to the protons entering the transmembrane domain and to their transient weak interaction with the protonatable residues therein.

We took the drug binding rate to be $k_{D}^{+}=r_{D}\nu_{D}$ is a characteristic timescale for a molecule to bind once in proximity to its binding site (as characterized by ν_*D*_). This describes the case where drug-pump association is dominated by diffusion. *r_D_* captures the effects of potential energy barriers between coarse-grained states [49], and the unbinding rate is $k_{D}^{-}=r_{D}e^{E_{D}∕k_{B}T}$. This ensures detailed balance relations are satisfied with respect to the drug concentration gradient [52].

Similarly for the protons, $k_{p}^{+}=r_{p}\nu_{p}$ and $k_{p}^{-}=r_{p}e^{E_{p}∕k_{B}T}$, with *r_p_* and ν_*p*_ defined analogously. While the physical interpretation of these quantities for proton binding is less clear, defining them provides the basis for exploring the entire parameter space of the kinetic model. We note that the forward and reverse rates for each step must satisfy *local* detailed balance relations, as set by the thermodynamic force relevant to the individual transition. This accounts, for instance, for the distinction between the factor $e^{E_{p}∕k_{B}T}$ appearing in $k_{p}^{-}$ and the factor *e^E_D_/k_B_T^* appearing in $k_{D}^{-}$. As a result, the entire system does not satisfy a global detailed balance relation, reflecting the fact that it operates away from equilibrium and gives rise to nonzero fluxes at steady state [53].

In the simplified model of Fig 2, the final transition from state 3 to 1 comprises four separate events. However, in spite of its complexity, the rate of this transition can be compactly related to differences in the energies of states 1 and 3 as follows:


$$\begin{array}{llll} k_{t}^{+} & =r_{t}\frac{e^{-q\Delta V∕k_{B}T}e^{-(E_{1}-E_{3})∕k_{B}T}}{1+e^{-q\Delta V∕k_{B}T}e^{-(E_{1}-E_{3})∕k_{B}T}}\; & & \;\\ & =r_{t}\frac{\nu_{D}\nu_{p}K_{D}K_{p}K_{G}}{1+\nu_{D}\nu_{p}K_{D}K_{p}K_{G}}.\; \end{array}$$ (4)

Since the system returns to its initial state after this step, it follows that $E_{1}\;-\;E_{3}=-E_{D}\;-\;E_{p}$, accounting for the change in internal energy. We can substitute these energy values for other parameters via Eqs. (2) and (3).  - *qΔV* is the change in the electrostatic energy of the protons upon translocation into the cytoplasm, and $K_{G}=\exp(-q\Delta V∕k_{B}T)$ is the associated Boltzmann factor. Eq. (4) reflects the fact that the electric potential acts during the conformational change, forcing the proton from the periplasm to the cytoplasm (we assume here that the drug molecule is uncharged). The form of the denominator ensures that the expected behaviour occurs in various limits (e.g. weak/strong binding, membrane potential).

The rate constant $k_{t}^{-}$ for reverse transitions 1 → 3 is then fixed by local detailed balance relations as [53,54]


$$\begin{array}{c} k_{t}^{-}=r_{t}\frac{\nu_{D}\nu_{p}}{1+\nu_{D}\nu_{p}K_{D}K_{p}K_{G}}. \end{array}$$ (5)

We note the presence of a free parameter *r_t_* with units of inverse time, analogous to *r_D_* and *r_p_* but without interpretation as the characteristic rate for any single process. It is necessary to capture microscopic details not explicitly accounted for in the model while providing the richness to explore the parameter space as needed. Its presence can be understood as a consequence of the simplicity of this three-state model, which we have chosen to employ with the hopes of achieving a high degree of generality in our analysis. Various elaborations upon the model to bring it more into line with a specific system may eliminate the need for this parameter; one such case is the richer model discussed below.

Eqs. (2)–(5), along with the relevant dependence of transition rates on drug molecule and proton concentrations, combine to form the following constraint on the entire cycle:


$$\begin{array}{c} \frac{k_{D}^{+}{\left[D\right]}_{in}}{k_{D}^{-}}\frac{k_{p}^{+}{\left[p\right]}_{per}}{k_{p}^{-}}\frac{k_{t}^{+}}{k_{t}^{-}{\left[D\right]}_{out}{\left[p\right]}_{cyt}}=K_{G}\frac{{\left[D\right]}_{in}{\left[p\right]}_{per}}{{\left[D\right]}_{out}{\left[p\right]}_{cyt}}=e^{-(\Delta \mu_{D}+\Delta \mu_{p})∕k_{B}T}, \end{array}$$ (6)

where $\Delta \mu_{D}=k_{B}T\ln({\left[D\right]}_{out}∕{\left[D\right]}_{in})$ is the chemical potential difference for drug molecules measured across the membrane from the inside to the outside of the cell and $\Delta \mu_{p}=q\Delta V\;+\;k_{B}T\ln\left({\left[p\right]}_{cyt}∕{\left[p\right]}_{per}\right)$ is the electrochemical potential difference for protons traveling from the periplasm into the cytoplasm. These definitions reflect that the proton is, of course, electrically charged, while we assume for simplicity that the drug molecule is not. $\Delta \mu_{D}\;+\;\Delta \mu_{p}$ amounts to the global free energy change associated with exactly one completion of the kinetic cycle, capturing the local detailed balance relations satisfied by the transition rates and ensuring their consistency with the Second Law of Thermodynamics [55].

The rate *J* of drug efflux generated by the pump at steady state can be calculated as the net population flux from state 3 to state 1 in the kinetic scheme:


$$\begin{array}{c} J=k_{t}^{+}P_{3}^{SS}-k_{t}^{-}{\left[D\right]}_{out}{\left[p\right]}_{cyt}P_{1}^{SS}. \end{array}$$ (7)

The steady-state populations $P_{1}^{SS}$ and $P_{3}^{SS}$ can be obtained from the system of Eqs. (1) by setting each time derivative Ṗ*_i_* to zero.

### Pump throughput is determined by more than just the drug binding affinity to the pump and the proton motive force

Upon solving the system of equations (1) in the steady-state condition and evaluating Eq. (7), the efflux rate *J* can be written in terms of the model parameters as,


$$\begin{array}{c} J=k_{t}^{+}\frac{{\left[D\right]}_{in}}{K_{M}+{\left[D\right]}_{in}}\frac{{\left[p\right]}_{per}}{K_{\beta }+{\left[p\right]}_{per}}\left(1-\frac{1}{K_{G}}\frac{{\left[D\right]}_{out}}{{\left[D\right]}_{in}}\frac{{\left[p\right]}_{cyt}}{{\left[p\right]}_{per}}\right). \end{array}$$ (8)

In the limit that $K_{G}\gg {\left[D\right]}_{out}{\left[p\right]}_{cyt}∕{\left[D\right]}_{in}{\left[p\right]}_{per}$, i.e., the *irreversible* limit, the factor in brackets tends to 1 and the efflux rate *J* described by Eq. (8) reproduces the experimentally observed Michaelis-Menten (MM)-like dependence of the efflux rate on the drug concentration [20,56].

*K_β_* and *K_M_* may be understood to be a pair of effective Michaelis-Menten constants. The former is given by


$$\begin{array}{c} K_{\beta }=K_{p}\left(1+\frac{r_{t}}{r_{p}}\frac{\nu_{D}K_{D}K_{G}}{1+\nu_{D}\nu_{p}K_{D}K_{p}K_{G}}\right). \end{array}$$ (9)

*K_M_* may be viewed as the effective affinity with which the pump extrudes drug molecules: the drug concentration at which the efflux is half of the value at which it will saturate, in analogy to binding/unbinding kinetics. It is a complicated function of the characteristic rates, binding affinities, electric potential, outside drug concentration, and both proton concentrations (S1 Text). Under experimentally relevant conditions (S1 Text), it is approximated as


$$\begin{array}{c} K_{M}\approx K_{D}\frac{\frac{r_{t}}{r_{D}}\nu_{p}K_{G}{\left[p\right]}_{per}+C}{\frac{{\left[p\right]}_{per}}{K_{p}}+C}, \end{array}$$ (10)

where $C=1+\nu_{D}K_{D}K_{G}(\frac{r_{t}}{r_{p}}+\nu_{p}K_{p})$.

Importantly, this effective affinity depends explicitly on the periplasmic concentration of the protons and the membrane potential, approaching the value of *K_D_* only in the limit of very low [p]_*per*_ and otherwise varying dramatically with the other environmental parameters. This reflects that, in general, the drug affinity towards the pump is not the only determinant of its specificity.

**Fig 3:**
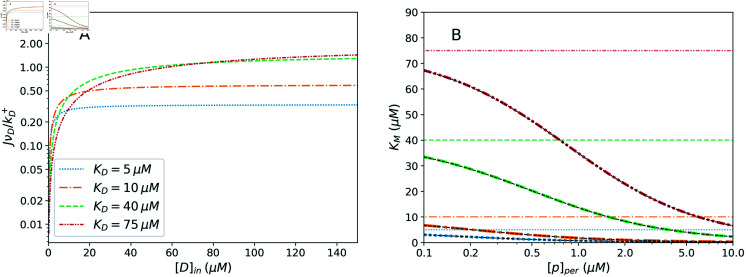
Quantities characterizing the efflux. A: The dimensionless efflux, $J\nu_{D}∕k_{D}^{+}$, as a function of [D]_*in*_ for varying *K_M_*. The efflux increases monotonically with [D]_*in*_, as predicted by Eq. (8) and consistent with Michaelis-Menten kinetics. The value of *K_D_* influences the drug concentration level at which the efflux rate begins to saturate, as well as the value to which it saturates. [*p*]*_per_* = 1 μ*M*. B: The Michaelis-Menten constant, *K_M_*, as a function of the periplasmic proton concentration. The narrow, black, dashed lines represent the simplified expression, Eq. (10), showing agreement with the exact values. The faded horizontal lines show each raw drug dissociation constant, *K_D_*, for comparison to the *K_M_* value characterizing the efflux. *K_M_* is approximated reasonably well by *K_D_* only in the limit of low [p]_*per*_, and otherwise varies with variations in the periplasmic pH, membrane voltage, and characteristic rates. [*D*]_*in*_ = *K_D_ = 10 μM*. In both panels, [*D*]_*out*_ = 10 μM and [*p*]_*cyt*_ = 0.1 μM. The remaining parameter values are set as *T* = 295*K*, *r_D_ = 10^8^ *s*^-1^, *r_p_* = 10^14^ *s*^-1^*, *r_t_* = 10^17^ *s*^-1^, *v_D_* = 1 *M*^-1^, *ν_p_* = 10^-6^ *M*^-1^, *K_p_* = 0.1 μM, and *K_G_* = 100 (S1 Text).

The dependence of the efflux on the inside drug concentration and *K_D_* is illustrated in Fig 3A which shows normalized non-dimensional efflux $J\nu_{D}∕k_{D}^{+}$. This represents the efflux rate relative to the characteristic rate for a drug molecule to diffuse to the binding site once in proximity, as characterized by ν_*D*_. The aforementioned Michaelis-Menten kinetics are demonstrated, with the efflux rate saturating as the drug concentration inside the cell increases. Since *K_G_* tends to be large, the bracketed factor in Eq. (8) is close to 1, corresponding to the irreversible limit of pump operation. Furthermore, the flux saturates to different levels for different *K_D_* values indicating the ability of the pump to discriminate between different molecules independent of their concentration, known as “absolute discrimination” [57,58].

Under the parameter values in the ranges discussed above, various simplifications (for example, *ν_D_ν_p_K_D_K_p_K_G_* ≪1) lead to the derivation of Eq. (10). One can see by inspecting this expression that, in this regime, *K_M_* is approximated reasonably well by the drug dissociation constant, *K_D_*, only in the limit of low periplasmic proton concentration. This behaviour is shown in Fig 3B–*K_M_* is seen to deviate from *K_D_* as the proton concentration in the periplasm grows.

A few insights concerning the factors affecting the efflux rate can be drawn here from the functional form of *J*. Focusing first on the dependence of the efflux on drug concentrations, we note that the factor in the brackets recovers the expected linear dependence on the chemical potential gradient, Δμ_*D*_, in the near-equilibrium regime [40], since


$$\begin{array}{c} \frac{{\left[D\right]}_{out}}{{\left[D\right]}_{in}}=e^{\Delta \mu_{D}∕k_{B}T}\approx 1+\frac{\Delta \mu_{D}}{k_{B}T} \end{array}$$ (11)

when Δμ_*D*_ ∕*k_B_ T* is small, characterizing the linear response regime. However, [D]_*in*_ also appears in the factor describing the MM-like behaviour, here as part of the ratio [*D*]_*in*_ ∕ *K_M_*. The effective affinity *K_M_*, in turn, depends strongly on the membrane potential, as well as the proton concentration gradient via its direct dependence on [p]_*per*_, outside of the special case that $r_{t}\nu_{p}K_{p}K_{G}∕r_{D}\approx 1$ (S1 Text). We stress that this is a direct manifestation of the nonequilibrium nature of the pump’s operation, distinct from the electrochemical gradients simply amounting to the thermodynamic forces driving the efflux. The complicated behaviour of *K_M_* stands in sharp contrast to any equilibrium situation, where the dissociation constant, *K_D_*, is the sole quantity relevant to characterizing the drug’s interaction with the pump. Accordingly, experimental validation that the model considered here captures genuine effects on the operation of efflux pumps would begin with verifying the Michaelis-Menten like dependence of the efflux on concentrations, and probing how the affinities themselves vary with each experimentally controllable parameter.

Turning attention to the effects of the proton concentrations, we identify the “proton motive force" (p.m.f.), -Δμ_*p*_, as the quantity most often assumed relevant to the operation of proton-powered molecular motors (with the negative sign as a consequence of conventions established when we introduced the model). As for the drug concentration gradient, the membrane potential and proton concentrations do appear in this form in the bracketed factor in Eq. (8), once again recovering the expected linear response behaviour close to equilibrium, namely, via the approximation,


$$\begin{array}{c} \frac{{\left[p\right]}_{cyt}}{{\left[p\right]}_{per}}\frac{1}{K_{G}}=e^{\Delta \mu_{p}∕k_{B}T}\approx 1+\frac{\Delta \mu_{p}}{k_{B}T}, \end{array}$$ (12)

and the neglecting of terms beyond first order in the thermodynamic forces. Moreover, the coefficient describing the linear dependence of the efflux on Δμ_*p*_ near equilibrium is equal to that describing its dependence on Δμ_*D*_, as a direct consequence of the Onsager reciprocity relations–that the dependence of the drug efflux on the proton gradient must match that of the proton flux on the drug concentration gradient–along with the fact that the proton flux is equal to the efflux due to the unicyclic nature of the model [40].

However, the periplasmic proton concentration has two other critical effects on the efflux rate. These are the Michaelis-Menten-like dependence characterized by affinity *K_β_*, and the aforementioned role of [p]_*per*_ in setting the MM constant for drug binding. These are both nontrivial effects that depend on [p]_*per*_ independently of its role in the electrochemical potential that drives the system away from equilibrium. These three distinct effects give rise to a complicated [p]_*per*_-dependence of the efflux. In general, they can oppose one another, making it difficult to predict, *a priori*, and highly dependent on the values of other parameters, how the efflux will change in response to a change in [p]_*per*_.

Finally, just like the proton concentrations, the electric potential difference *ΔV* enters into the equation for *J* not only through the proton motive force. *K_G_* appears on its own in the definition of *K_M_*, as well as Eqs. (4) and (5) for the rates of the final, multi-step transition. This dependence is also present only away from equilibrium. It ceases in the linear response regime, since *K_G_* is the Boltzmann factor associated with the membrane potential, so it may be taken to 1 as a valid leading-order approximation near equilibrium.

### Varying the periplasmic pH shifts the optimal *K_D_* range for efficient pumping

**Fig 4:**
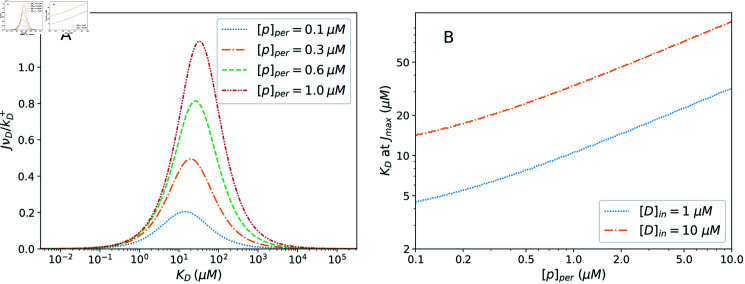
Impacts of proton concentration on efflux pump operation. A: Dimensionless efflux rate, $J\nu_{D}∕k_{D}^{+}$, as a function of the drug dissociation constant *K_D_* for varying values of [p]_*per*_. The efflux is peaked in a particular *K_D_* range and suppressed on either side of the peak. [*D*]_*in*_ = 10 μ*M*. B: The value of the drug dissociation constant, *K_D_*, at which the efflux rate is maximized, through a range of periplasmic proton concentrations. Other parameter values in both panels are the same as in Fig 3.

A key insight offered by this model of efflux pump kinetics is revealed when examining how the efflux rate varies as a function of the drug dissociation constant, *K_D_*, for different values of the periplasmic pH. Each curve in Fig 4A shows how the efflux varies over a broad range of *K_D_* values. We see that, for a given set of parameter values, the efflux peaks at a particular value of *K_D_* and dies off for both very weakly and very strongly binding molecules. The position of this peak is determined by competing effects of the proton motive force and drug binding affinity.

Physically, drugs that bind too weakly (high *K_D_*), are likely to unbind from the cavity before the subsequent proton binding can take place. Thus, they cannot proceed to the final transition that sees the drug transported across the membrane. For too strong binding, however, the rate $k_{t}^{+}$ for the final transition that involves the unbinding of the drug into the exit duct is greatly suppressed due to its dependence on *K_D_*, as in Eq. (4), limiting the overall rate at which the drug efflux can proceed.

We have established that the efflux rate exhibits MM-like behaviour with respect to the concentration of protons in the cytoplasm. This accounts for the different peak heights between the flux curves in Fig 4A for different periplasmic proton concentrations. However, these curves also reveal secondary effects of varying [p]_*per*_. Namely, through its influence on the Michaelis-Menten constant, *K_M_*, changes to [p]_*per*_ lead to changes in the values of *K_D_* at which the efflux is maximized. Specifically, in the regime studied, the optimal value of *K_D_* for efflux shifts higher for higher periplasmic proton concentration (lower pH). This shifting effect is visible in Fig 4A, though somewhat obscured by the logarithmic scale of the horizontal axis. It is emphasized in Fig 4B, where the *K_D_* value maximizing *J* is shown to shift through a factor of about 5 as the periplasmic pH varies from 7 to 5. This is understood as a consequence of the fact that increases to [p]_*per*_ speed up the 2 → 3 transition towards state 3. The time spent in state 2 is reduced, diminishing the concern that a weak binding drug will detach prematurely, and amplifying the benefits of its ability to readily detach and exit the cell *after* the conformational transition.

The predictions of this model indicate that the periplasmic pH, amongst its other roles, may play a role in modulating the response of the efflux pump to a particular drug used in treatment. In other words, different periplasmic pH conditions may be optimal for the transport of different types of drugs, offering an explanation of the flexibility of efflux pumps to transport a variety of different molecules.

In addition to the shifting of the peak, increases to the periplasmic proton concentration grow the overall size of the *K_D_* range at which the efflux is significant. In particular, as seen in Fig 4A, it extends this range to higher values of *K_D_*, representing weaker binding molecules. The high *K_D_* range is precisely where self molecules would be expected to be found, as the cell would be unlikely to have a pump that strongly binds the self molecules it wishes to retain. Thus, high [p]_*per*_ may increase the risk of extruding self molecules, to detrimental effect, but may still be favourable overall in the presence of a relatively weakly binding drug. Notably, even at high [p]_*per*_, the peak is still considerably narrower than that of a proton-independent passive transporter operating under comparable conditions (S1 Text). That is, in addition to driving the pump and tuning the range of *K_D_* values at which it is most effective, the presence of a proton gradient-dependent transition helps the pump to achieve greater specificity overall.

### Specificity tuning behaviour extends to a more detailed model

The three-state model introduced above captures the minimal energetic and kinetic features of the pump and allows the derivation of the analytic expression for the efflux, Eq. (8). This serves as a basis for the analysis of the factors affecting efflux pumps’ tunability and identification of the principles of their specificity. However, by grouping multiple steps that include conformational changes, drug unbinding, and proton unbinding into one final transition, it effectively makes the assumption that these molecular processes are all very strongly coupled and occur at a rate determined by the slowest process among them. This model also does not capture the possibility for the drug and proton binding affinities to differ with the conformation of the transporter.

**Fig 5:**
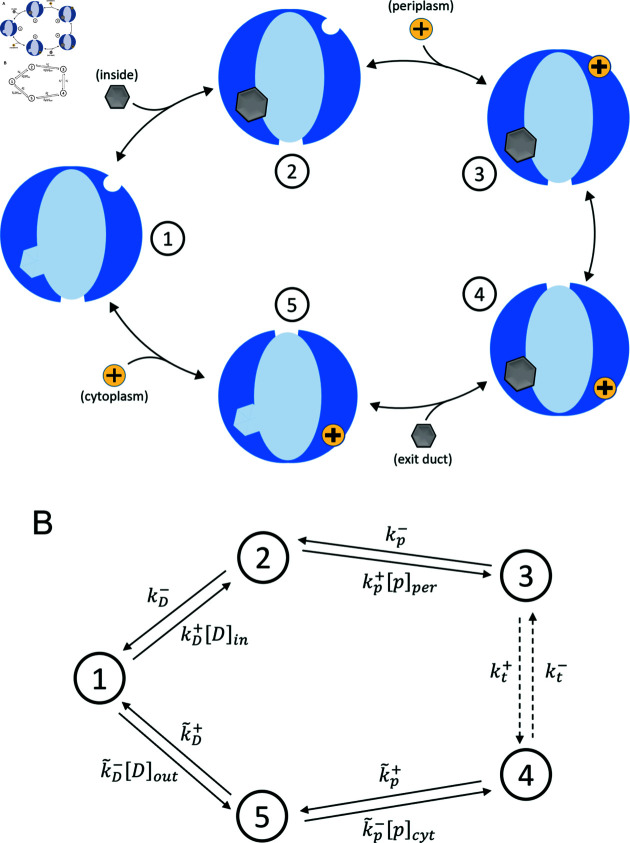
Kinetic scheme for the five-state model. A: Five-state model for efflux pump operation. In contrast to the three-state model, the first conformational change, drug unbinding to the exit duct, proton unbinding to the cytoplasm (with return to the initial conformation) are split into separate steps. B: The five-state model represented as a directed graph with edges corresponding to transitions. Each transition is labelled with its respective rate, as they arise in the master equation (S1 Text).

In addition to the three state model, we studied a more complete kinetic scheme which consists of five states, treating the conformational change exposing the drug to the exit duct, drug unbinding to the exit duct, and proton unbinding to the cytoplasm as three separate transitions, each with a distinct forward and reverse rate. This model is depicted in Fig 5A, where states 1-3 are equivalent to the states of the three-state model. In state 4, the conformational change of the pump has occurred such that the proton binding site now faces the cytoplasm and the interior of the active transporter now opens towards the exit duct, but both the drug and the proton remain bound. In state 5, the drug molecule has been released while the proton remains in place. In the final transition from state 5 back to state 1, the proton is released to the cytoplasm, and the pump, in the absence of a proton responding to the membrane electric potential, promptly returns to its initial conformation. Fig 5B depicts each transition as a directed edge, labelled by its rate.

This model is described using the master equation formalism, analogous to Eqs. (1), with a set of differential equations for the probabilities for the system to be found in each of the five states (S1 Text). The forward and reverse rate constants, $k_{D}^{+}$, $k_{D}^{-}$, $k_{p}^{+}$, and $k_{p}^{-}$ for the transitions between states 1, 2, and 3, are unchanged from the three-state model, along with the dissociation constants *K_D_* and *K_p_* for drug binding from the cytoplasm, and protons from the periplasm, respectively.

The additional flexibility offered by the five-state model, however, allows one to consider how the dissociation constant (and thus, binding energy) for drug molecules may differ in different conformational states of the transporter. That is, the dissociation constant, ${\tilde{K}}_{D}=e^{{\tilde{E}}_{D}∕k_{B}T}∕\nu_{D}$, for drug molecules when the pump has undergone a conformational transition and the binding site is exposed to the exit duct is, in general, not equal to *K_D_*. The same is true for protons and is modelled by the introduction of ${\tilde{K}}_{p}$ and Ẽ_*p*_, the dissociation constant and binding energy for protons from the cytoplasm. As another additional parameter, we may consider the energy change, $E_{c}\equiv E_{4}-E_{3}$, that occurs during the first conformational transition alone. The model is constrained only by the requirement that the system returns to its initial state upon completion of the cycle, so,


$$\begin{array}{c} E_{D}+E_{p}+E_{c}-{Ẽ}_{D}-{Ẽ}_{p}=0, \end{array}$$ (13)

Assuming that the characteristic rates, *r_D_* and *r_p_*, and interaction volumes, ν_*D*_ and ν_*p*_, are the same as above, the forward rate for the transition from state 4 to 5 is ${\tilde{k}}_{D}^{+}=r_{D}\nu_{D}{\tilde{K}}_{D}$ with the reverse rate ${\tilde{k}}_{D}^{-}{\left[D\right]}_{out}=r_{D}\nu_{D}{\left[D\right]}_{out}$. Similarly, the forward rate for the transition from state 5 to state 1 is ${\tilde{k}}_{p}^{+}=r_{p}\nu_{p}{\tilde{K}}_{p}$, with the reverse rate ${\tilde{k}}_{p}^{-}=r_{p}\nu_{p}{\left[p\right]}_{cyt}$. Equation (13) then dictates that *E_c_* is given by


$$\begin{array}{c} E_{c}=k_{B}T\ln\left(\frac{{\tilde{K}}_{D}{\tilde{K}}_{p}}{K_{D}K_{p}}\right). \end{array}$$ (14)

Local detailed balance relations determine the forward and reverse rate constants for the conformational transition between states 3 and 4,


$$\begin{array}{llll} k_{c}^{+} & =r_{c}\frac{e^{-E_{c}∕k_{B}T}e^{-q\Delta V∕k_{B}T}}{1+e^{-E_{c}∕k_{B}T}e^{-q\Delta V∕k_{B}T}}\; & & \;\\ & =r_{c}\frac{1}{1+\frac{1}{K_{G}}\frac{{\tilde{K}}_{D}{\tilde{K}}_{p}}{K_{D}K_{p}}},\; \end{array}$$ (15)

and


$$\begin{array}{c} k_{c}^{-}=r_{c}\frac{1}{1+K_{G}\frac{K_{D}K_{p}}{{\tilde{K}}_{D}{\tilde{K}}_{p}}}, \end{array}$$ (16)

where we have introduced *r_c_*, a characteristic rate for the conformational change between states 3 and 4. This is a more physically meaningful quantity than its counterpart, *r_t_*, in the three-state model, suggesting that moving to five states leads to a more realistic model with less need for fine-tuning. The factor of *K_G_* in $k_{c}^{+}$ reflects that the electric potential acts on the proton during this step of the cycle, forcing it from an area of high potential (as its binding site is exposed to the periplasm) to an area of lower potential (the cytoplasm). However, it remains bound throughout this step.

**Fig 6:**
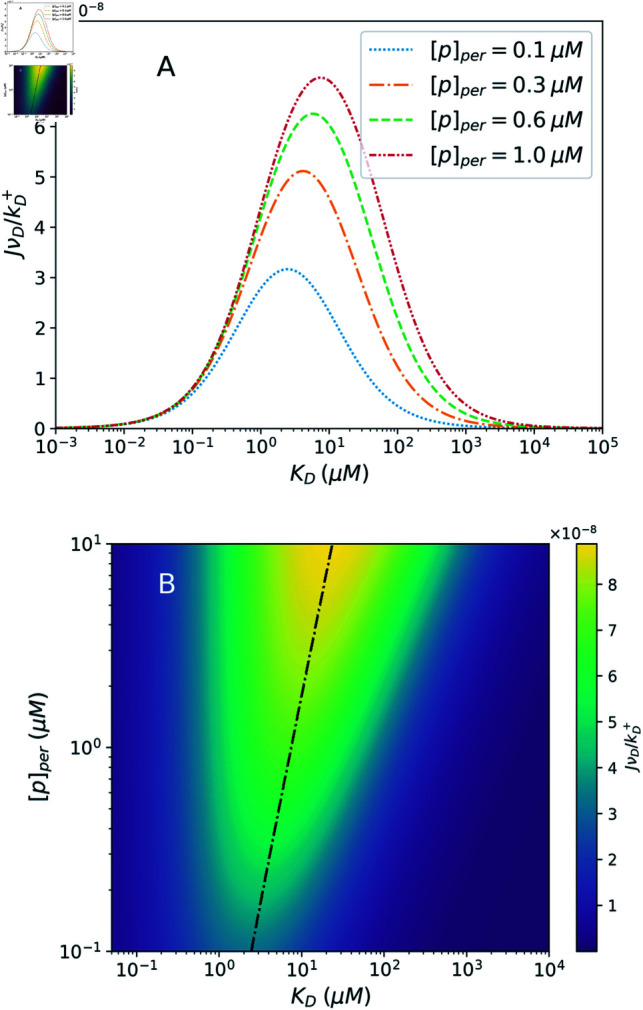
Numerical results for the five-state model. A: Dimensionless efflux rate given by the five-state model as a function of the drug dissociation constant, *K_D_*, of drug binding from the cytoplasm. Four different values of the periplasmic proton concentration, [p]_*per*_, are shown. Similar to the three-state model, maximum efflux occurs within a particular *K_D_* range which shifts towards higher values of *K_D_* with increasing [p]_*per*_. B: Contour plot showing the variation of the dimensionless efflux rate through the range of periplasmic pH values from 7 to 5. The dash-dotted line represents the value of *K_D_* at which the efflux rate is maximized for each value of [p]_*per*_. In both panels, we assume that drug molecules bind less strongly from the outside of the cell: ${\tilde{K}}_{D}∕K_{D}=10$. $K_{p}={\tilde{K}}_{p}=0.1\;\mu M$, *r_c_* = 10^6^ *s*^-1^, and parameter values are otherwise the same as in Fig 4.

The five-state model does not simplify to provide analytic insights. Therefore, we obtained the efflux rate by numerically solving the system of equations at steady state. Efflux is plotted against *K_D_* in Fig 6A, showing the same qualitative behaviours seen with the three-state model: maximal efflux at a particular *K_D_* range which shifts higher with increases to [p]_*per*_. These results are shown as a contour plot in Fig 6B, over the periplasmic pH range from 7 to 5.

This model exhibits the peak-shifting effect with varying [p]_*per*_ to a similar degree to the three-state model. As seen in Fig 6, the *K_D_* value for maximum efflux shifts through a factor of about 5 as the periplasmic pH drops from 7 to 5. For the figure, it is assumed that ${\tilde{K}}_{D}∕K_{D}=10$, i.e., drugs bind to the transporter with greater affinity when it is in its initial conformation, with the drug binding site facing the interior, than when it is in its second conformation with the binding site facing the exit duct. This would be a feature of a pump that is particularly effective at extruding unwanted molecules from the cell, though the peak-shifting effect is still observed with this assumption relaxed.

We note that we considered the behaviour of the pump using the five-state model only in a particular region of a very large parameter space, determined based on experimental relevance, but without optimization. If the parameter values were to be optimized to maximize the peak-shifting effect and/or the specificity of the pump, the increased parameter space may allow even better performance to be achieved with the five-state model than with the three-state model.

## Discussion

Bacterial efflux pumps are known to play a prominent role in the development of resistance to a broad range of antibiotics. We have studied kinetic models that capture important features of efflux pump operation to elucidate unexpected nonequilibrium effects at play in governing their behaviour. The resulting insights suggest a potential mechanism by which multidrug resistance can arise.

Past studies have utilized kinetic models to help in understanding the translocation process of antibiotics and other molecules within active transporters [10,20,27,28,59] as well as non-specific transmembrane pores [60]. Most of the published kinetic models are Michaelis-Menten like in their description of kinetics. Importantly, those describing proton-powered active efflux pumps inherently assumed that pumps are driven by the proton motive force and did not consider explicitly the distinct effects of varying the proton concentrations and membrane gradient. Many others considered ATP-powered transporters instead.

We have shown, through analysis using a set of minimal models that explicitly take into account the two separate components of the proton motive force–the membrane potential and the proton concentration gradient–that the operation of cellular efflux pumps exhibits complex and nontrivial dependence on these nonequilibrium electrochemical gradients. It is characterized by behaviour that resembles Michaelis-Menten kinetics. However, there is an additional factor resulting from the microscopic reversibility of the process and there are effective nonequilibrium Michaelis-Menten constants that differ from expected equilibrium values, which are dictated solely by the binding affinities. These affinities can be modulated by varying the periplasmic proton concentration so as to shift the range of drug dissociation constants at which the pump operates most effectively, offering variability as to how effectively the pump can extrude any given drug. Furthermore, the transporter extrudes molecules over a narrower range of *K_D_* values overall than a passive channel. These effects may help explain what underlies the applicability of efflux pumps towards a wide range of different drug molecules, while they can still maintain a good degree of specificity towards their target.

Until now, common approaches to the inhibition of efflux pumps comprise the targeting of the efflux pump expression, disruption of the pump self-assembly process, design of new antibiotic molecules that are not recognized by efflux pumps, and new competing substrates to block pump binding sites [1,7,17,61–64]. In spite of the research efforts, only a limited number of inhibitors are currently in the pre-clinical stage and there are no inhibitor drugs for bacterial efflux pumps in clinical use [61]. The effects of periplasmic pH on pump operation, including the ability to tune their specificity towards molecules in a particular range of binding affinities, may be a consideration as researchers aim to develop treatments for bacterial infections that target the operation of these pumps.

We have characterized the phenomenon of specificity tuning through alteration of the periplasmic pH via the peak-shifting effect demonstrated in Figs 4 and 6. An additional effect of increasing the periplasmic proton concentration is reduced specificity, or an overall larger range of *K_D_* values at which the efflux pump throughput is relatively high (S1 Text). This increases the risk of extruding other cytoplasmic contents, which are expected to be weak binders, characterized by a high *K_D_* value. In the presence of drug molecules, the various factors affecting efflux pump operation may be optimized to best address this trade-off, shifting the efflux peak to target drug molecules, while keeping the pump as specific to the drug as possible.

To emphasize the scope of the proton concentration’s ability to tune efflux behaviour, we considered the linear response regime for the three-state model (S1 Text). In this case, all thermodynamic forces–the chemical potential differences due to the concentration gradients and the membrane voltage–are small enough relative to the thermal energy that we may Taylor-expand only to linear order in each. To good approximation, the efflux is given by,


$$\begin{array}{c} J_{LR}=k_{t}^{+,eq}\frac{{\left[D\right]}_{in}}{K_{M}^{eq}+{\left[D\right]}_{in}}\frac{{\left[p\right]}_{per}}{K_{\beta }^{eq}+{\left[p\right]}_{per}}\left(-\frac{\Delta \mu_{p}+\Delta \mu_{D}}{k_{B}T}\right), \end{array}$$ (17)

where the superscript *eq* denotes the equilibrium value of each constant, calculated by setting *K_G_* = 1, [*D*]_*out*_ = [*D*]_*in*_, and [*p*]_*cyt*_ = [*p*]_*per*_. Δμ_*D*_ and Δμ_*p*_ are defined as below Eq. (6). Interestingly, in this regime, while the effect of the proton *gradient* is limited to the linear dependence expressed in Eq. (17), the efflux still depends in an absolute sense on the proton concentration, which scales the overall efflux rate via Michaelis-Menten kinetics and plays a role in setting the equilibrium affinity, $K_{M}^{\mathrm{eq}}$.

Another quantity to consider when assessing the efflux pump performance is the entropy production rate. In the absence of external sources, the concentrations of the protons and drugs will not remain constant, and the system will evolve towards chemical equilibrium. For our models, we have assumed instead that concentrations of drug molecules and protons are kept constant to maintain non-zero efflux. This requires continuous input (and dissipation of energy). This energetic cost can be understood by implicitly assuming that independent translocation processes occur that transport protons and drug molecules back across the membrane to maintain their gradients. Each of these requires a minimum energy input per drug/proton, determined by the change in the chemical potential differences of each in this auxiliary process: -Δμ_*D*_ and -Δμ_*p*_, respectively (we note that if the drug concentration is higher outside the cell than inside, -Δμ_*D*_ is negative and the gradient-maintaining process can simply amount to passive transport). Accordingly, the minimal energy dissipation to maintain the steady state non-zero efflux is *JΔF*, where


$$\begin{array}{c} \Delta F=-\Delta \mu_{D}-\Delta \mu_{p}=k_{B}T\ln({\left[D\right]}_{in}∕{\left[D\right]}_{out})+k_{B}T\ln({\left[p\right]}_{per}∕{\left[p\right]}_{cyt})-q\Delta V. \end{array}$$ (18)

Since our models impose a strict one-to-one ratio between the number of drugs extruded and the number of protons transported across the membrane, one can only determine a lower bound on the entropy production rate that is simply proportional to the efflux, *J*. They therefore describe a simple tradeoff relation between pump throughput and dissipation: the greater the efflux, the higher the minimum rate of dissipation necessary to maintain the gradient.

In order to go beyond this simple case, one can augment the kinetic model with the addition of a “futile” cycle, wherein a proton is transported into the cytoplasm, but no drug molecule is extruded (S1 Text). In this case, the lower bound on the entropy production rate exceeds that predicted from the three- and five-state models, as it must account for the additional proton flux that occurs without any corresponding drug efflux. In incorporating such a futile cycle, however, there is no improvement to the tunability of the pump, and only a minor improvement to the specificity of the efflux pump due to the fact that the futile cycle allows for greater suppression of the efflux at high *K_D_*. We did not identify any clear tradeoff relation between specificity and dissipation, distinguishing this study from other works which aim to uncover the constraints thermodynamics places on various measures of precision in cellular processes [65–67].

Conversely, we did find that this model exhibits a greater degree of specificity than a passive channel whose operation is completely independent of the proton electrochemical gradient. This suggests that a role is played by the external nonequilibrium driving force in achieving a certain level of pump performance (S1 Text).

In this study, we focused on the hitherto unappreciated nonequilibrium effects of the periplasmic pH on the pump throughput. We considered variations in the drug binding affinity and the periplasmic pH as two independent variables, assuming one can be held fixed through a range of values of the other. For practical applications it may be important to consider the direct effects of the pH on the equilibrium binding affinities *K_D_* and *K_p_* [68,69], and investigate whether this enhances or reduces the specificity and tunability effects outlined in Results.

In summary, we studied a set of new quantitative dynamical models for bacterial efflux pump active transporters that explicitly take into account the separate components of the proton motive force, membrane potential and the proton gradient. Our models show that, unlike the commonly held assumptions, the proton concentration gradient does not merely provide the thermodynamic force to drive the pump; it also takes part in determining the pump selectivity. Furthermore, the roles of its two components–the proton concentration gradient and the electric potential gradient across the membrane–enter this determination separately. Thus, modifications to each independently play a role in tuning the efflux mechanism. These results build upon prior understanding of the role that alteration of the free energy landscape characterizing a biological process can play in modulating fluxes [49].

The structure of the model and the main ideas behind its formulation are in line with a number of detailed modeling studies of the Na^+^/glucose cotransporter [25,46,47], as well as resistance-nodulation-division pumps such as AcrAB found in *E. coli* and MexAB in *P. aeruginosa* [14–19]. This new role of the electrochemical gradient can, in principle, be relevant to other types of ion-driven active transporters as well.

## Methods

### Master equation formalism

The master equations that we used to describe the time evolution of the probability for the system to be in each state (e.g., for the three-state model, Eq. (1)) can be used to calculate the efflux directly. This probability distribution at any given time is represented by a vector $\vec{P}(t)$ whose dimension is equal to the number of states in the kinetic model. The equations of motion may then be written as a matrix-vector product


$$\dot{\vec{P}}=M\vec{P},$$

where each element *M_ij_* is the rate to transition from state *j* to state *i* and the diagonal elements are set such that all columns sum to zero, ensuring that ∑⁡_*i*_ *P_i_* = 1 at all times.

Notably, we are interested in the behaviour of the system at steady state, ignoring any changes to the environmental parameters that would affect the matrix *M* on the basis that they would occur on a timescale much longer than the time taken for the probabilities to reach their steady-state values. This amounts to the probability distribution ${\vec{P}}^{SS}$ at which ${\dot{\vec{P}}}^{SS}=0$, which is equivalent to the normalized eigenvector of *M* associated with the zero eigenvalue.

Equipped with ${\vec{P}}^{SS}$ in terms of the model parameters, it is straightforward to calculate the net flux through any given transition. The efflux is identified as the sum of the net fluxes through each transition during which a drug molecule is released to the exit duct. It is, therefore, given by Eq. (7) for the three-state model, as the 2 → 3 transition is the only transition that includes the extrusion of a drug molecule. For a unicyclic model such as those considered here, the net flux through any transition will suffice, since the steady state condition guarantees all these are equal. When considering multicyclic schemes (S1 Text), care must be taken in identifying which transitions in the model are associated with the extrusion of drug molecules.

This process for calculating the efflux may be carried out numerically, i.e., by diagonalizing the matrix *M* after the input of numerical values for each parameter. For the three-state model, we were able to do it symbolically as well. This led, after algebraic manipulations, to the derivation of the expression for the efflux rate, Eq. (8), and the associated expressions for the effective affinities.

The identification of the *K_D_* value at which the efflux reaches its maximum, as plotted in Figs 4B and 6B, was done numerically, by identifying zero-crossings in the numerical derivatives (finite differences) of *J* as a function of *K_D_* for a range of [p]_*per*_ values.

### Graphics

All graphs were generated using Matplotlib with the results of calculations carried out by Python programs. The diagrams of the pump and kinetic cycles were created in Microsoft Powerpoint.

## Supporting information

S1 Text This file contains sections A–G, covering additional topics including more general analytic expressions, the linear response regime, determination of parameter values used in numerics, and more detail on additional models of membrane transporters. (PDF)
